# Supplementary figures and images for: Microglia integration into human midbrain organoids leads to increased neuronal maturation and functionality
Source: Glia. 2022 Mar 9;70(7):1267–88. doi: 10.1002/glia.24167 (PMC9314680; doi:10.1002/glia.24167)

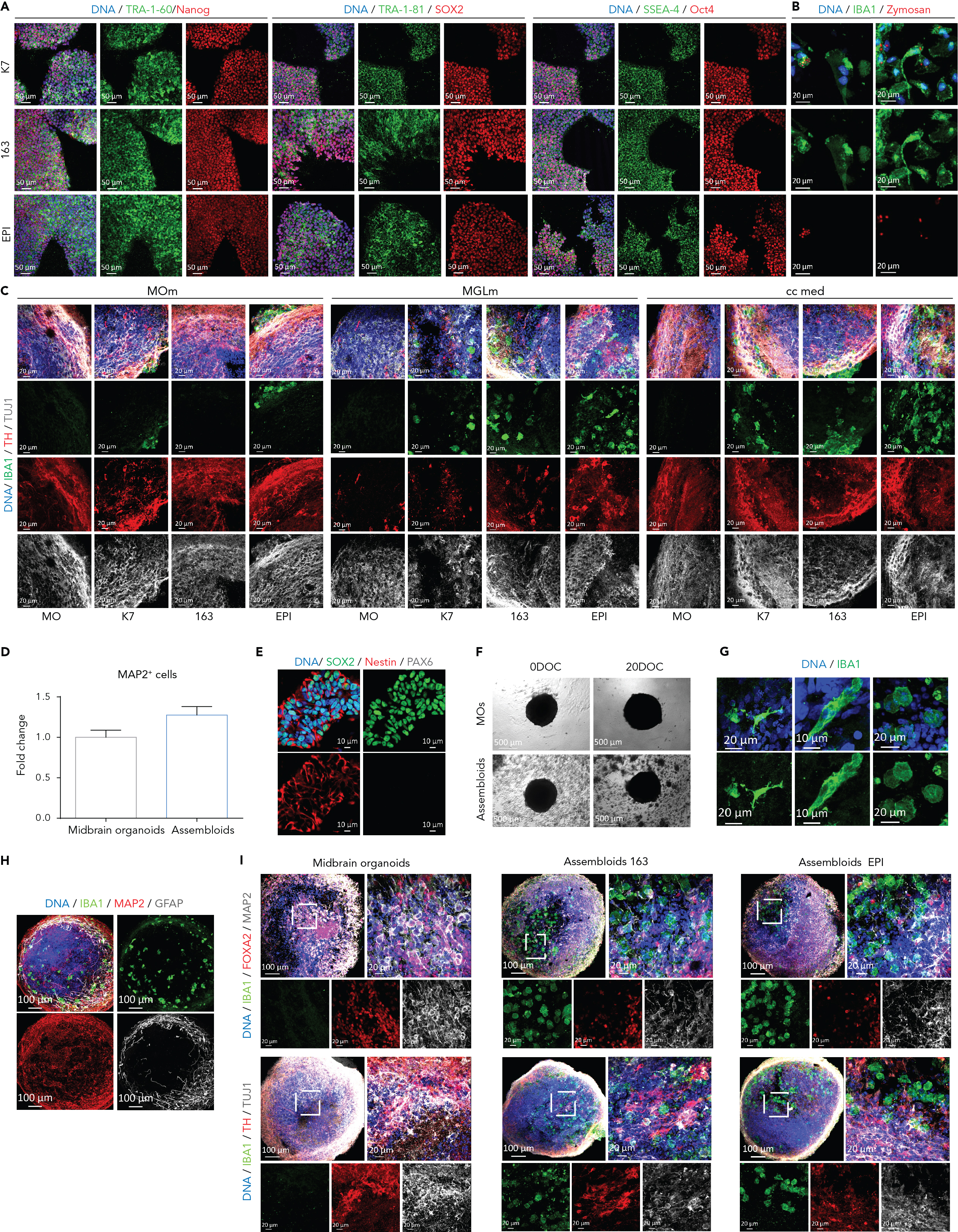

Supplement: Supplementary file 2 — Figure S1 Microglia in assembloids show different morphologies and allow astrocyte differentiation A. iPSCs from the line K7 (upper row), 163 (middle row) and EPI (bottom row) stained for the pluripotency markers TRA‐1‐60, Nanog (left), TRA‐1‐81, SOX2 (middle), SSEA‐4 and Oct‐4 (right). B. Zymosan and IBA1 staining on microglia from line K7 (top) and 163 (bottom) differentiated for 10 days. C. Immunostaining for IBA1, TH and TUJ1 of midbrain organoids and assembloids with microglia from line K7, 163 and EPI upon culture with midbrain organoid (MOm), microglia (MGLm) or coculture (cc med) media). D. MAP2 positive (MAP2+) cells in midbrain organoids and assembloids (n (midbrain organoids) =5, 5 batches, n (assembloids) =15, 5 batches, 3 cell lines). For immunofluorescence images see Figure 2C and S1H. Data are represented as mean ± SEM. Y axis is fold change compared with midbrain organoids. E. Immunostaining of neural precursor cells from line K7 for the pluripotency marker SOX2, the neural precursor marker Nestin and the forebrain and hindbrain marker PAX6, whose absence confirms the midbrain patterning of the cells. F. Bright field images of midbrain organoids (upper panels) and assembloids (bottom panels) at the coculture day (0DOC, days of coculture, left) and at 20DOC (right). G. Immunostaining of assembloids for IBA1 showing ramified (left), elongated (middle) and round (right) microglia. H. Immunostaining of an assembloid from the line 163 after 70 days of coculture, for GFAP, IBA1 and MAP2. I. Immunofluorescence staining of midbrain organoids (left panels) and assembloids with microglia from line 163 (middle panels) and EPI (right panels) for IBA1, FOXA2 and MAP2 (upper panels), and for TH and TUJ1 (bottom panels). The images correspond to a maximum intensity projection from a z‐stack of 70 μm organoid sections. [file GLIA-70-1267-s005.tif]

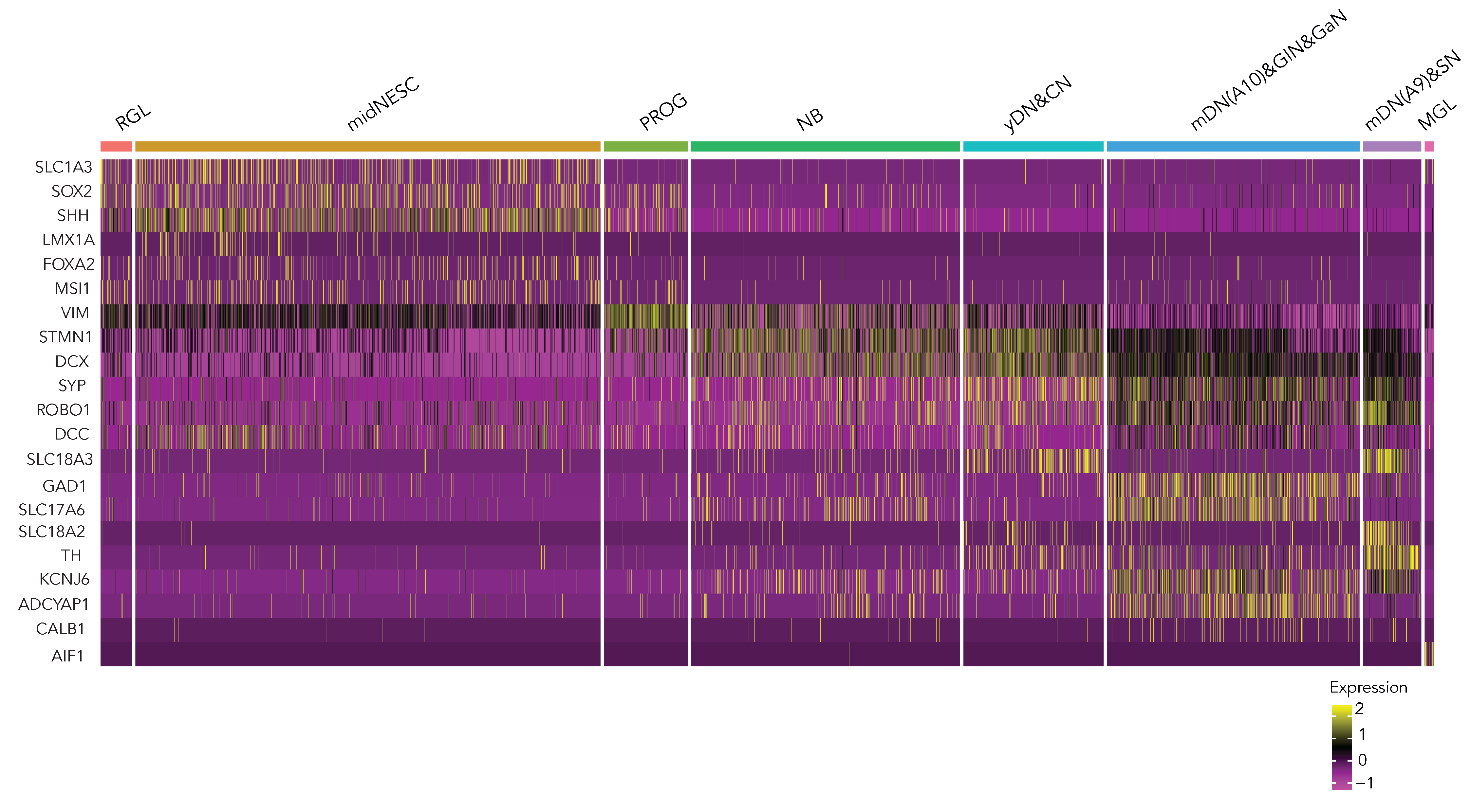

Supplement: Supplementary file 3 — Figure S2 Cell type specific gene expression in assembloids. Heatmap showing the cell type‐specific gene expression throughout the different cell clusters in assembloids. [file GLIA-70-1267-s009.tif]

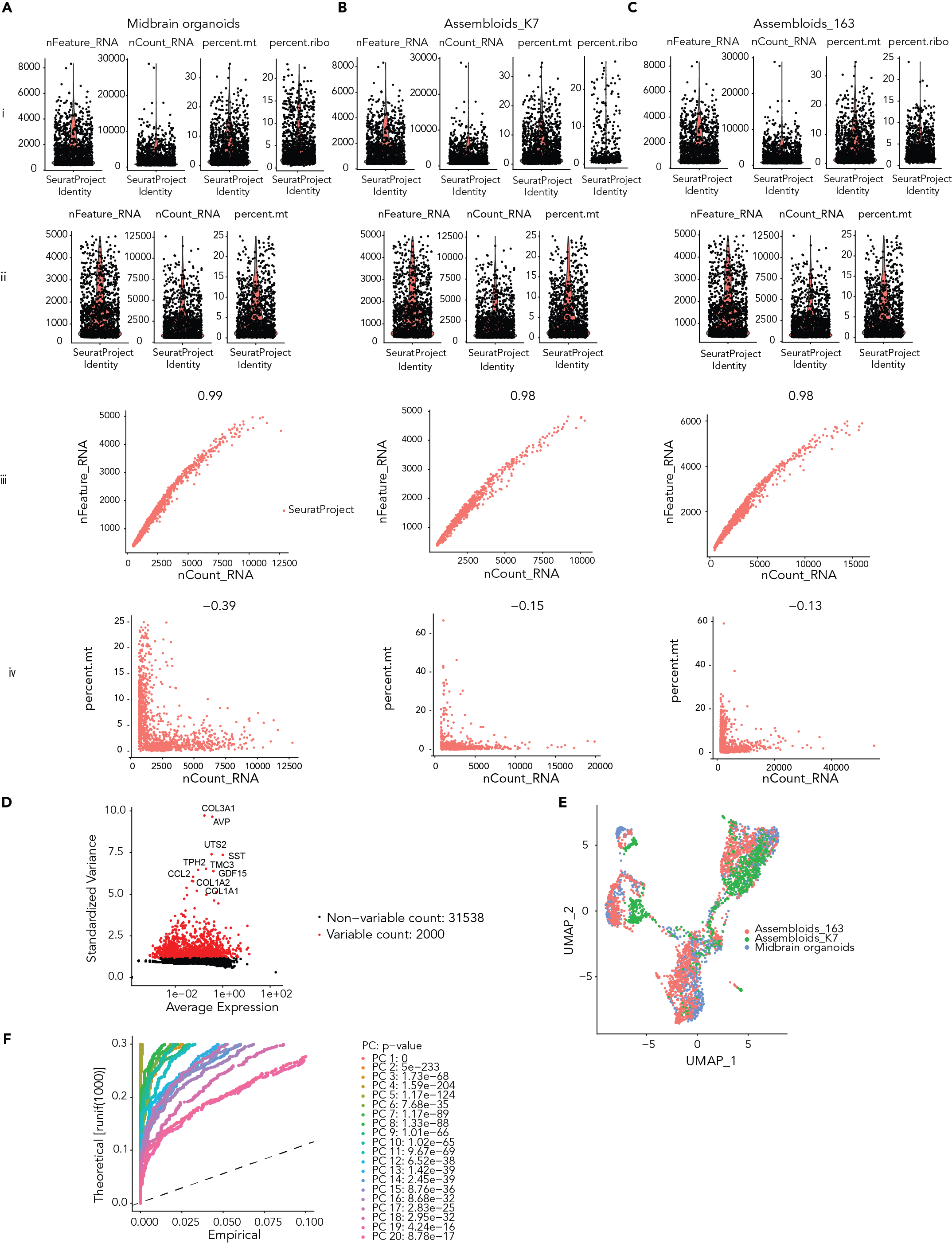

Supplement: Supplementary file 4 — Figure S3 Quality control of sn‐RNAseq data using the Seurat R Package. Quality controls of A. Midbrain organoids. B. Assembloids with microglia from line K7. C. Assembloids with microglia from line 163. i) Before quality controls; ii) After quality controls (100 < nFeature_RNA < 5000 or percent_mt < 25); iii) Correlation between features and counts; iv) Correlation between microglial genes and counts. D. Volcano plot showing the most variable genes between midbrain organoids and assembloids. E. UMAP visualization of scRNA‐seq data grouped by samples: Assembloids with microglia from 163 Assembloids_163), Assembloids with microglia from K7 (Assembloids_K7) and midbrain organoids. F. Principle components analysis. 20 dimensions were chosen. [file GLIA-70-1267-s012.tif]

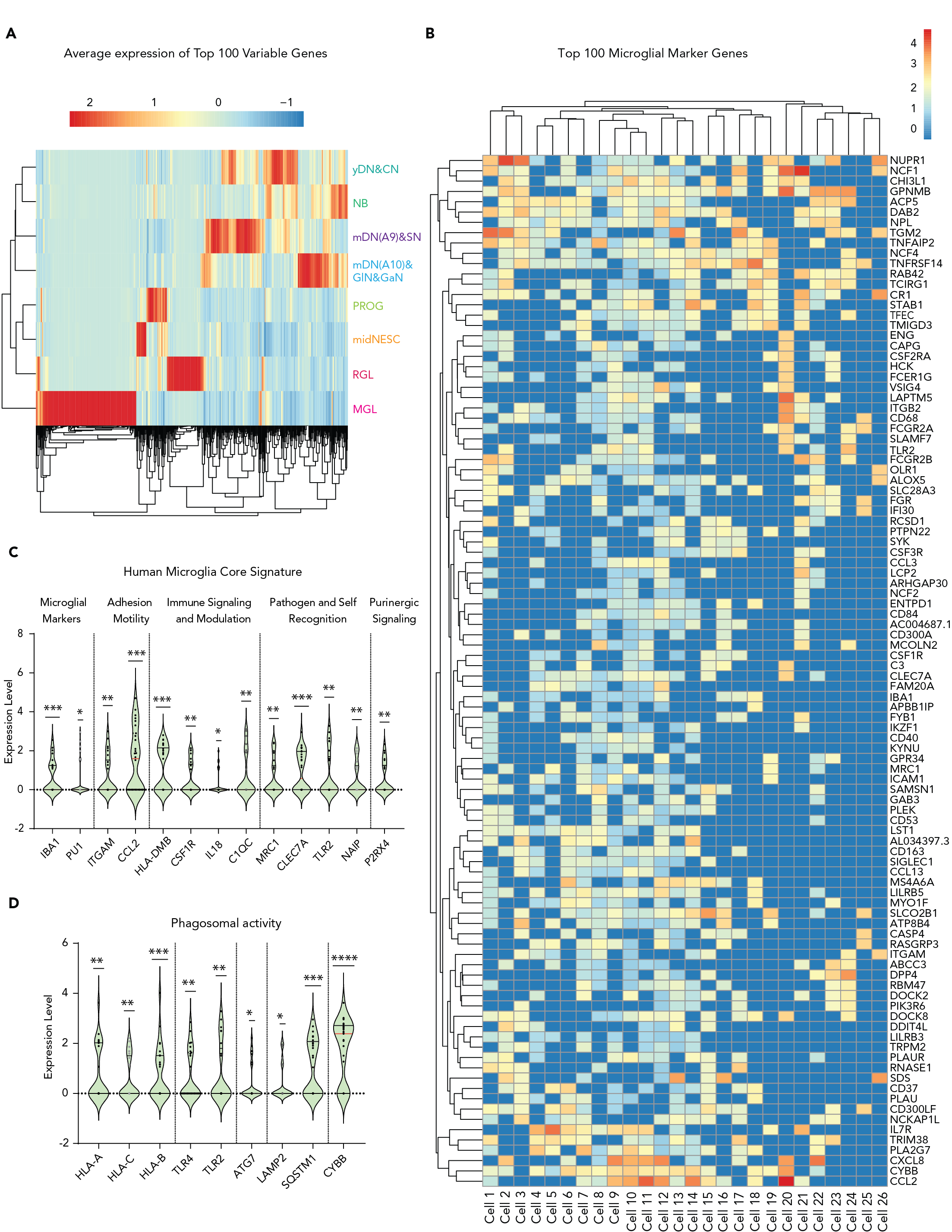

Supplement: Supplementary file 5 — Figure S4 Microglia in assembloids have a typical immune cell signature. A. Heatmap showing the average expression of 100 most variable genes between midbrain organoids and assembloids. B. Expression of top 100 microglial marker genes across microglia cells in assembloids. C. Microglia core signature, expression of microglia marker genes as well as genes involved in adhesion, immune response, pathogen recognition and purinergic signaling. D. Gene expression levels of genes related to phagocytic activity. Violin plot shows average expression level. *p <0.05, **p <0.01, ***p <0.001, ****p <0.0001 using a Wilcox one‐sample tests, median in red, quantiles in black. Dots represent single cells. [file GLIA-70-1267-s011.tif]

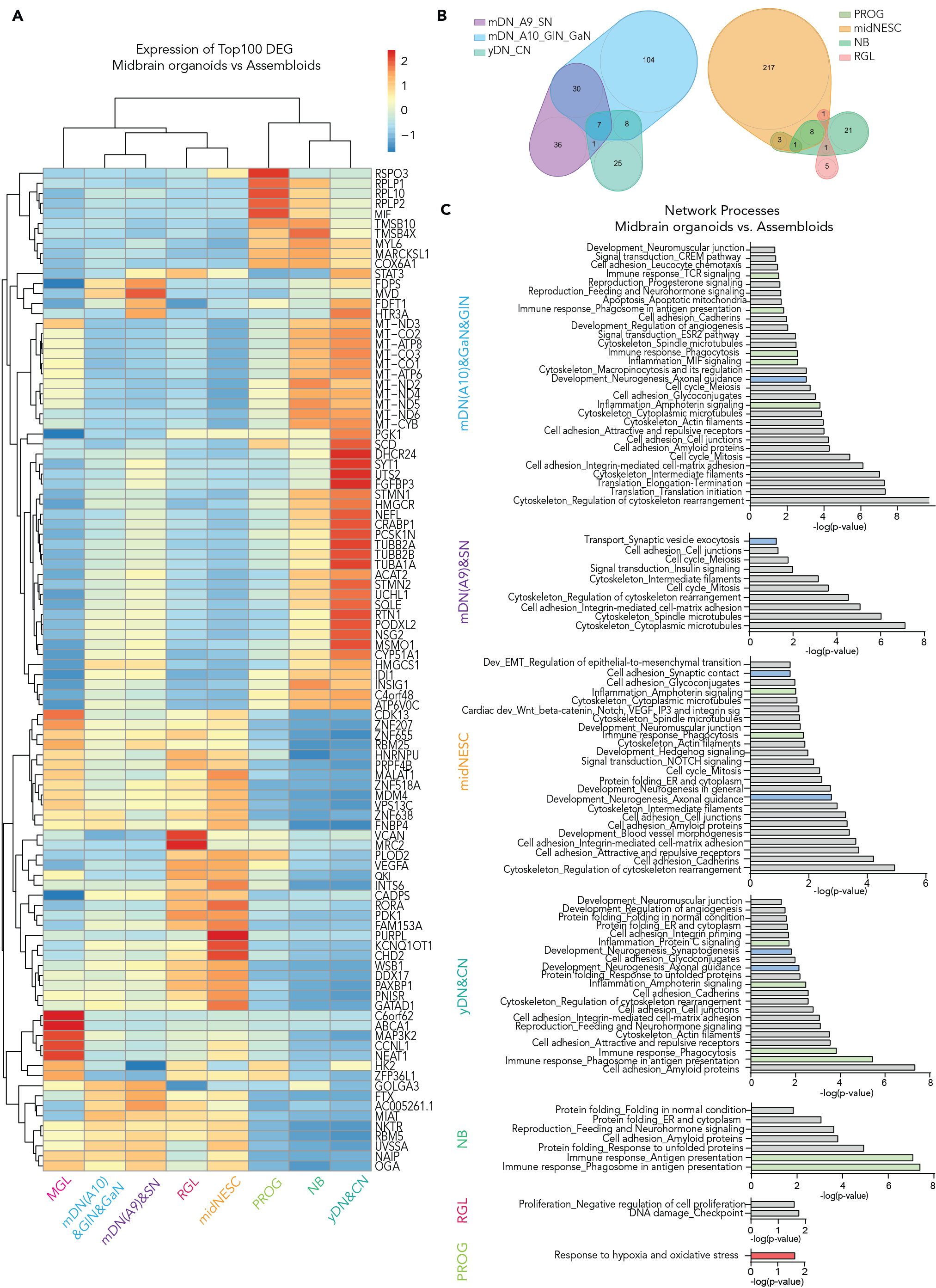

Supplement: Supplementary file 6 — Figure S5 Microglia in assembloids lead to differential expression of multiple genes. A. Heatmap of the average expression of 100 most significant differentially expressed genes across cell clusters in midbrain organoids and assembloids (p <0.05). B. Venn diagrams showing the number of DEG across cell types. Overlap within three neuronal clusters (left pannel) and midNESC, PROG, RGL and NB (right panel). C. Complete enrichment analysis of cluster specific DEG between midbrain organoids and assembloids reveals significant network processes (FDR <0.05). [file GLIA-70-1267-s008.tif]

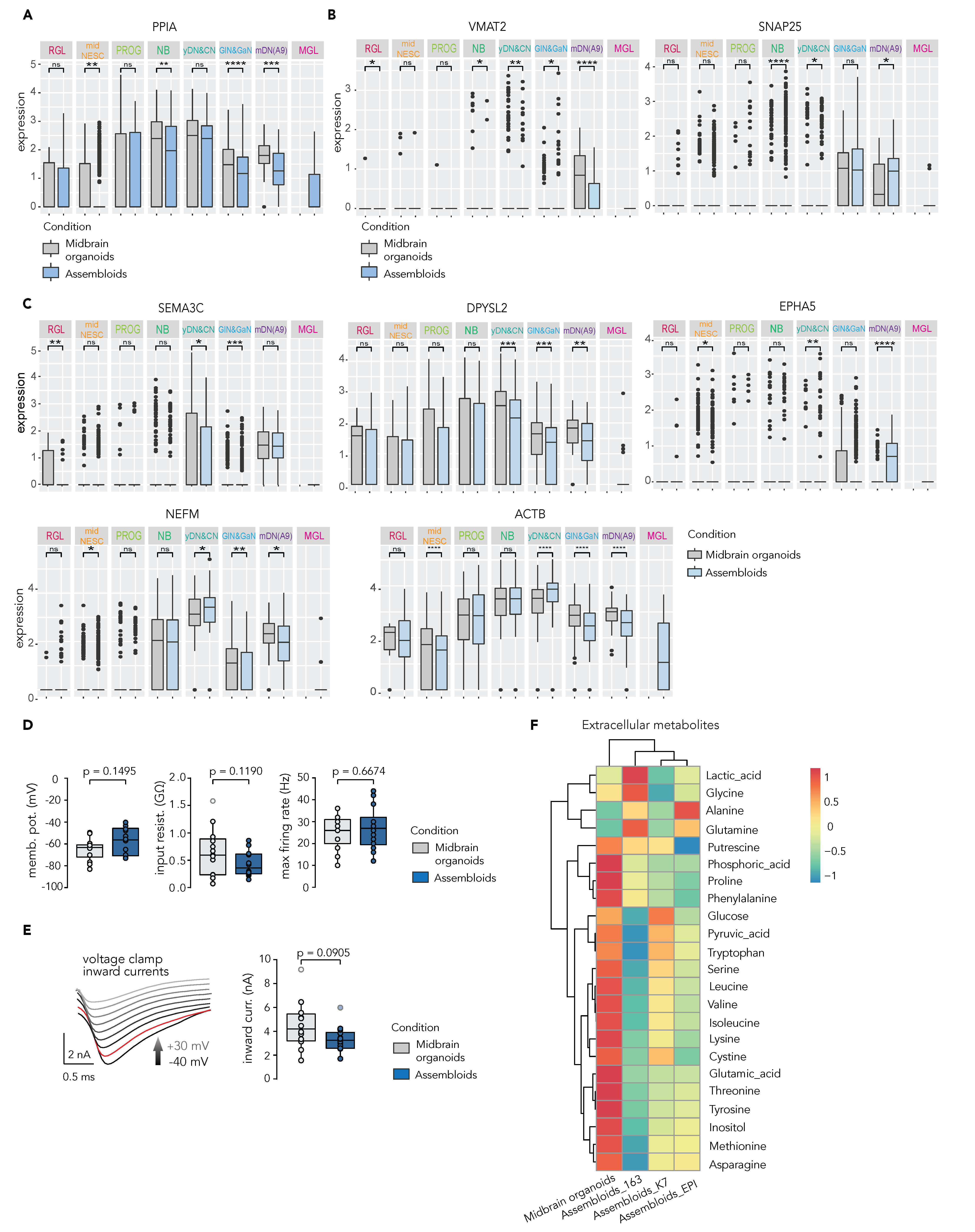

Supplement: Supplementary file 7 — Figure S6 Microglia lead to a decrease in the inflammasome‐related gene PPIA, and differences in synapse‐related genes. A. Expression of PPIA gene, involved in pyroptosis, across cell clusters in midbrain organoids and assembloids. B. Expression of VMAT2 and SNAP25, involved in synaptic vesicle exocytosis, across cell clusters in midbrain organoids and assembloids. C. Expression levels of axonal guidance and growth‐related genes: semaphorins (SEMA3C, DPYSL2), plexins, ephrins (EPHA5), neuropilins, neurofilaments and actin cytoskeleton (NEFM, ACTB) across cell clusters in midbrain organoids and assembloids. Data are represented as mean ± SD. * p <0.05 using a Wilcox test. Dots represent single cells. D. Boxplot from patch clamp data showing that neurons in midbrain organoids and assembloids show similar resting membrane potentials (left) and input resistances (middle), and fired repetitive action potentials in response to somatic current injections (right, n midbrain organoids =14; n assembloids = 13 cells). E. Inward currents of an assembloid neuron triggered by voltage steps to different potentials starting from −70 mV in voltage‐clamp mode. Voltage‐gated currents appeared at ‐40 mV and persisted until +30 mV under whole‐cell voltage‐clamp conditions (left). Inward currents generated at −30 mV (red trace in left panel) between midbrain organoids and assembloids showed no significant difference between both groups (right). F. Heatmap showing extracellular metabolite levels in culture supernatants (n (midbrain organoids) = 5, 5 batches, n (assembloids) = 15, 5 batches, 3 cell lines). Midbrain organoids cluster separately from assembloids from lines K7, 163 and EPI. [file GLIA-70-1267-s002.tif]

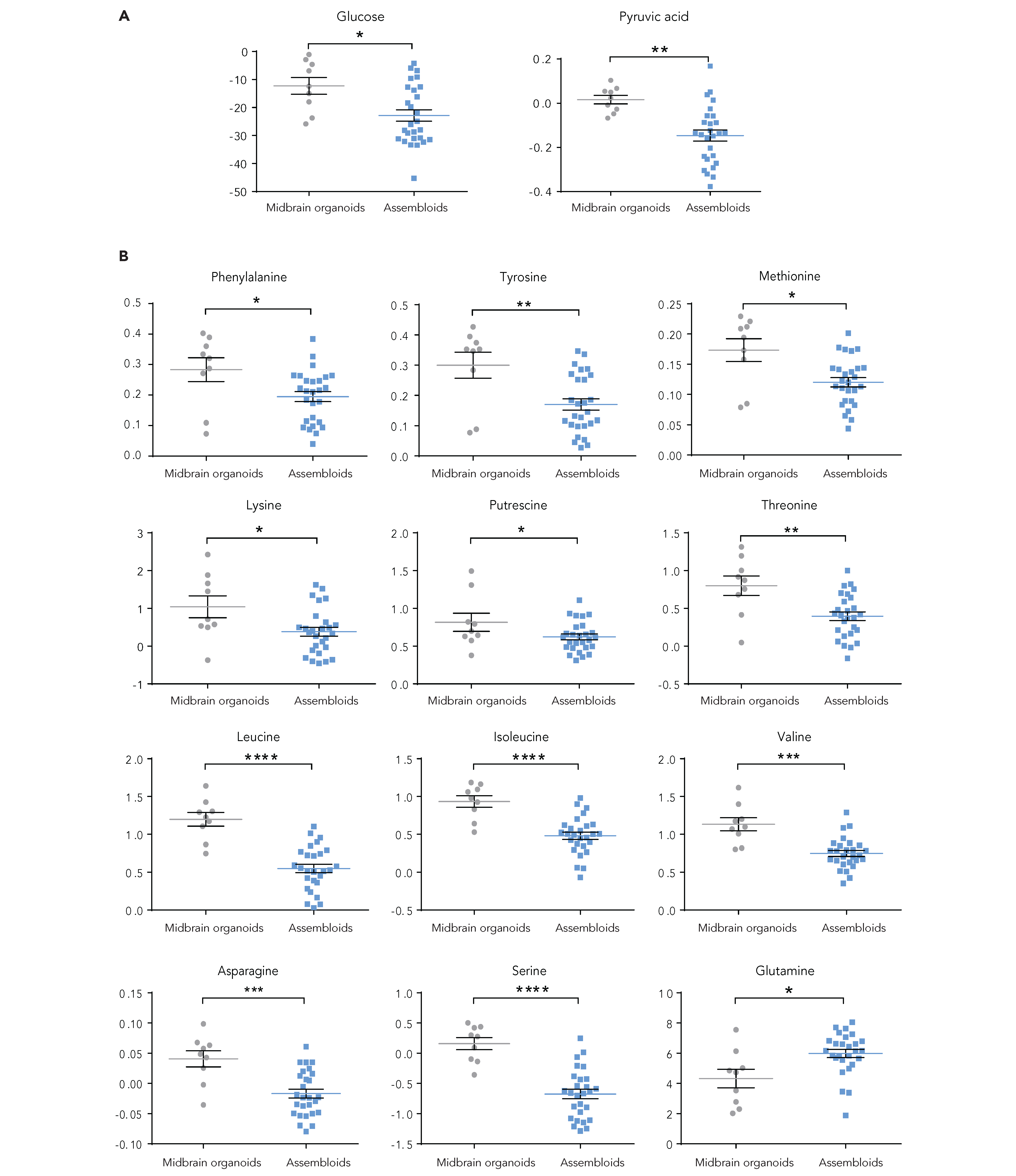

Supplement: Supplementary file 8 — Figure S7 Assembloids show a different extracellular metabolite profile compared with midbrain organoids. A. Metabolite levels in the culture media from organoids and assembloids after 48 h of culture. The levels of glucose and pyruvic acid were lower in media from assembloids compared with midbrain organoids. B. The levels of the amino acids phenylalanine, tyrosine, methionine, lysine, putrescine, threonine, leucine, isoleucine, valine, asparagine and serine in the media were lower in assembloids, whereas the glutamate levels were higher (n (midbrain organoids) = 3, 3 batches, n(assembloids) = 9, 3 batches, 3 cell lines). Each dot represents a replicate (medium 3 organoids or assembloids pooled). Data are represented as mean ± SEM. *p <0.05, **p <0.01, ***p <0.001, ****p <0.0001 using a Mann–Whitney or an unpaired t test. [file GLIA-70-1267-s007.tif]
